# High-Intensity Exercise Reduces Cardiac Fibrosis and Hypertrophy but Does Not Restore the Nitroso-Redox Imbalance in Diabetic Cardiomyopathy

**DOI:** 10.1155/2017/7921363

**Published:** 2017-06-18

**Authors:** Ulises Novoa, Diego Arauna, Marisol Moran, Madelaine Nuñez, Sebastián Zagmutt, Sergio Saldivia, Cristian Valdes, Jorge Villaseñor, Carmen Gloria Zambrano, Daniel R. Gonzalez

**Affiliations:** ^1^Department of Basic Biomedical Sciences, Faculty of Health Sciences, Universidad de Talca, Talca, Chile; ^2^Institute for Chemistry of Natural Resources, Universidad de Talca, Talca, Chile; ^3^Department of Human Movement Sciences, Faculty of Health Sciences, Universidad de Talca, Talca, Chile

## Abstract

Diabetic cardiomyopathy refers to the manifestations in the heart as a result of altered glucose homeostasis, reflected as fibrosis, cellular hypertrophy, increased oxidative stress, and apoptosis, leading to ventricular dysfunction. Since physical exercise has been indicated as cardioprotective, we tested the hypothesis that high-intensity exercise training could reverse the cardiac maladaptations produced by diabetes. For this, diabetes was induced in rats by a single dose of alloxan. Diabetic rats were randomly assigned to a sedentary group or submitted to a program of exercise on a treadmill for 4 weeks at 80% of maximal performance. Another group of normoglycemic rats was used as control. Diabetic rat hearts presented cardiomyocyte hypertrophy and interstitial fibrosis. Chronic exercise reduced both parameters but increased apoptosis. Diabetes increased the myocardial levels of the mRNA and proteins of NADPH oxidases NOX2 and NOX4. These altered levels were not reduced by exercise. Diabetes also increased the level of uncoupled endothelial nitric oxide synthase (eNOS) that was not reversed by exercise. Finally, diabetic rats showed a lower degree of phosphorylated phospholamban and reduced levels of SERCA2 that were not restored by high-intensity exercise. These results suggest that high-intensity chronic exercise was able to reverse remodeling in the diabetic heart but was unable to restore the nitroso-redox imbalance imposed by diabetes.

## 1. Introduction

Diabetes mellitus is one of the most common chronic diseases all over the world, becoming an epidemic, triggered probably by reduced physical activity and increased obesity in the population [1, 2]. Diabetic cardiomyopathy is the deterioration of the myocardial function and morphology produced by the altered glucose homeostasis imposed in diabetes, independent of coronary disease [3]. Diabetic cardiomyopathy is characterized initially by diastolic dysfunction and cardiac hypertrophy, with preserved ejection fraction. At the cellular level, the diabetic myocardium presents myocyte hypertrophy, interstitial fibrosis, and apoptosis [4]. This process of cardiac deterioration involves the generation of reactive oxidative species (ROS) [5]. Oxidative stress exists when the production of ROS outweighs their degradation by antioxidant systems [6]. The resultant elevation of ROS has numerous harmful effects on the cardiovascular system via cellular damage by oxidation, disruption of vascular homeostasis through interference with NO, and detrimental intracellular signaling pathways [7]. In a variety of animal models of diabetes and humans with diabetic cardiomyopathy, there is excessive ROS production from both mitochondrial and extramitochondrial sources, and ROS have been implicated in all stages of the development of heart failure, from cardiac hypertrophy to fibrosis, contractile dysfunction, and failure [5, 8].

Several ROS sources contribute to the observed oxidative stress in the diabetic heart such as xanthine oxidoreductase (XOR) [9], nicotinamide adenine dinucleotide phosphate (NADPH) oxidases (NOX) [10], mitochondria [9], and uncoupled nitric oxide synthases (NOS) [10–13].

A direct consequence of the increased production of reactive oxygen species (ROS) is NOS uncoupling. This is the aftermath of the oxidation of tetrahydrobiopterin (BH_4_), an essential cofactor for NOS activity. When NOS is uncoupled, its activity is redirected towards the production of superoxide, instead of nitric oxide (NO), further contributing to the oxidative process.

Physical exercise is prescribed as part of the rehabilitation for patients with heart diseases since it reduces cardiac risk factors, protects against myocardial damage, and improves cardiac function [14]. In type 2 diabetic patients, exercise is also advised as part of the nonpharmacological treatment, since it exerts a number of benefits such as improved insulin sensitivity and reduction in body weight [15]. Nevertheless, there is less information regarding type 1 diabetic patients. A recent report indicates that intense exercise is associated to reduction in the risk of cardiovascular events in type 1 diabetes patients [16]. In animal models of type 1 diabetes, low-to-moderate exercise training has been found to improve cardiac glucose metabolism [17], reduce apoptosis [18], and improve ventricular function [19].

The intensity of exercise for diabetic patients is a matter of intense investigation, being suggested that in particular, high-intensity interval training may confer additional cardiometabolic protection [20]. For instance, while moderate-intensity exercise did not improved cardiac function in type 2 diabetic patients [21], a similar study but with a high-intensity interval training showed improved cardiac function and remodeling [22]. Another study showed no difference between moderate- and high-intensity exercises [23], although this later did not include the assessment of cardiac parameters.

As mentioned before, in animal models of type 1 diabetes, low- and moderate-intensity exercise have been reported to reverse or prevent some of the cardiac maladaptations of diabetic cardiomyopathy [18, 24]. Nevertheless, the information regarding high-intensity exercise in diabetic cardiomyopathy is less abundant.

The mechanisms by which exercise may produce it beneficial effects include an increase in nitric oxide production [25] and a reduction in oxidative stress [26]. In this work, we tested the hypothesis that a high-intensity exercise training program could reverse the cardiac maladaptations and oxidative stress that are produced by diabetes.

## 2. Methods

### 2.1. Animals and Training Protocol

Diabetes was induced in 3-month-old male Sprague–Dawley rats by a single dose of alloxan (Sigma-Aldrich, St. Louis, MO), 200 mg/kg, intraperitoneal. Six days day after alloxan injection, hyperglycemia was confirmed (plasma glucose levels >300 mg/dL). Diabetic rats were randomly assigned to a sedentary group (*n* = 5) or submitted to a program of exercise on a motor-driven treadmill, 5 days/week, for 4 weeks. The maximal intensity of the training for each rat was assessed by stepwise increases in the treadmill speed. Two days prior to the test, the animals were submitted to a period of acclimatization in the treadmill, walking at 0.6 km/h, twice a day, the first day. During the second and third days, the incremental velocity test was applied that consisted of increasing the treadmill velocity from 0.6 km/h to 0.2 km/h every 3 min, with no upper limit, until the animal reached exhaustion that defines the end of the test. The angle of the treadmill was kept constant at 0°. Once the animal reached its maximal velocity for at least 3 minutes, this value was assigned as the maximal performance (100%). Then, the animal was trained at the 80% of its capacity (velocity and time), once a day, five days a week, for four weeks. Every week, maximal capacity was reevaluated for each animal, to adjust its training load for the next week.

To determine the duration (in minutes) of the training, a tolerance test was applied that consisted in determining the maximal time that the rat was able to sustain the 80% of the velocity previously determined. Finally, the training consisted of the 80% of the maximal velocity for the 80% of maximal time. This finally determines the volume of training for each animal. The rats were maintained in the animal facility of the Universidad de Talca, with food and water ad libitum, at room temperature (22 ± 5°C) and with cycles of 12 hrs light/darkness. The diet used was obtained from Champion® (20.5% crude protein, 5% fiber, 4% fat). A group of normoglycemic rats was used as control (*n* = 7). Finally, the rats were euthanized after two days after the training protocol finished, to avoid potential confounding effects of acute exercise. To extract the heart, the animals were induced deep anesthesia with ketamine (75 mg/100 g body weight) and xylazine (15 mg/100 g body weight), checking for the complete absence of sensitive reflexes.

Plasma glucose determinations were performed using the glucose oxidase system (Valtek Diagnostics, Santiago, Chile), following the manufacturer instructions, and using a spectrophotometer (Rayleigh UV-9200).

All procedures were performed in conform to the *NIH Guide for the Care and Use of Laboratory Animals*. The protocol was approved by the Bioethical Committee of the Universidad de Talca.

### 2.2. Histological Staining

After excision, hearts were fixed in 4% paraformaldehyde. After fixation, the samples underwent a series of dehydrations and were embedded in paraffin blocks. After this, 5 *μ*m sections were obtained with a Microm HM 325 microtome and then mounted on xylanized slides. Sections of hydrated and deparaffinized xylene tissue were stained with hematoxylin and eosin (H&E), used for TUNEL analysis or Sirius red staining.

### 2.3. Assessment of Cardiac Apoptosis

Apoptosis was evaluated by the TUNEL assay [27], which detects fragmented DNA in situ in the cell nucleus. The assay was performed using the TUNEL Apoptosis Detection Kit (EMD Millipore, Temecula, CA), according to the manufacturer's instructions with some modifications. Cardiac sections prepared as above indicated, then incubated with 50 *μ*L of proteinase K for 30 min in a humid chamber and washed with PBS. Then, sections were incubated with the TdT end-labeling cocktail that contains TdT and biotinylated dUTP, for 5 min at room temperature. Each step was followed by PBS washes. Then, sections were incubated with blocking buffer for 20 min at room temperature. After this, FITC-labeled avidin was applied for 30 min at 37°C in a humid chamber. Sections were washed twice with PBS for 15 min at room temperature in the dark and then counterstained with propidium iodide (1 : 2000) for 15 min. Sections treated with DNAse I was used as positive controls. Sections in which treatment with proteinase K was replaced by PBS were used as negative control. After finishing the protocol, sections were observed with a Zeiss LSM-700 confocal microscope and TUNEL positive cells and total cells were counted. The apoptotic index represents the number of TUNEL^+^ cells to total number of cells. This percentage was compared between groups.

### 2.4. Real-Time PCR

For quantitative polymerase chain reaction (qPCR), total RNA was extracted from rats hearts using TRIzol reagent and reversed transcribed using high capacity cDNA reverse transcription kit (Applied Biosystems, Foster City, CA). qPCR was performed in triplicate for each heart, using a 20 *μ*L mixture containing 1 ng cDNA, TaqMan Master Mix, and TaqMan® Gene Expression Assays (Applied Biosystems) for NOX2 (Rn00576710_m1) and NOX4 (Rn00585380_m1). As an internal control, glyceraldehyde 3-phosphate dehydrogenase GAPDH (Rn01775763_g1) was determined in each reaction. Reaction conditions were set according to the manufacturer: one cycle of 50° for 2 min, one cycle of 90° for 10 min, and 40 cycles of 15 s at 95° and 60° for 1 min using an Mx3000P qPCR system (Agilent Technologies, CA, USA). Relative fold change was calculated by the 2^ΔCt^ method and compared with baseline values as previously described [28].

### 2.5. Biopterin Measurements

Tetrahydrobiopterin (BH_4_) and dihydrobiopterin (BH_2_) were measured form cardiac homogenates, following the procedure described by Fukushima and Nixon [29], with modifications, by HPLC (Perkin Elmer series 200) separation and fluorescence detection at 350 nm (Shimadzu RF-20A). The procedure involves a differential oxidation BH_4_ and BH_2_ with iodine in acidic and basic conditions. In acidic conditions, both BH_4_ and BH_2_ are oxidized to biopterins, while under basic conditions, only BH_2_ is oxidized to biopterin. The difference in the content of biopterin between both oxidations represents the amount of BH_4_. Intracardiac BH_4_ content was normalized to the total protein content of the sample.

### 2.6. Western Blotting

Cardiac tissue was homogenized in 3 mL of lysis buffer (Tris 50 mM, SDS 0.1%, NaCl 30 mM, EDTA 2 mM) supplemented with 30 *μ*L proteases inhibitors cocktail (MP Biomedicals, Solon, OH) using an ultraturrax, as previously described [30]. Then, the homogenate was centrifuged at 4000 rpm for 10 min at 4°C. The supernatant was removed and protein concentration was quantified using the BCA method (BCA Protein Assay Kit, Thermo Fisher Scientific, Rockford, IL). For this, 100 *μ*g of protein were mixed with Laemmli buffer, separated by SDS-PAGE, and blotted onto nitrocellulose membranes (Bio-Rad Laboratories, Hercules, CA). Antibodies used were the following: for NOX2 (anti-gp91^phox^ 1 : 1000, catalog number: 611415, Lot: 15660), p67^phox^ (1 : 1000, catalog number: 610913, Lot: 000079141) and eNOS (1 : 2000, catalog number: 610297, Lot: 35170) from BD Biosciences (San Jose, CA); for NOX4, from Thermo Scientific (1 : 1000, catalog number: PA1-46014), serine 16 phosphorylated phospholamban (1 : 1000, catalog number: A010-12, Lot: 0214-01) and total phospholamban (1 : 1000, catalog number: A101-14, Lot: 642016) were obtained from Badrilla (Leeds, UK); SERCA2 (1 : 2000, catalog number: SC 8094, Lot: C1115); GAPDH (1: 1000, catalog number: SC-365062, Lot: A2715); and secondary antibodies from Santa Cruz (Santa Cruz, CA). The protein bands were visualized using Supersignal West Femto Reagent (Pierce, Rockford, IL). Western blots were scanned and bands were quantified by densitometry analysis using ImageJ software.

### 2.7. Statistical Analysis

Results are expressed as mean ± standard error. Comparisons between groups were performed using one-way analysis of variance (ANOVA) with Newman-Keuls and Dunnett as post hoc analysis, for data with normal distribution. For nonparametric data, Kruskal-Wallis test was applied, with Dunn's multiple comparisons test as post hoc analysis. Analysis of exercise training was performed using ANOVA with repeated measures. Statistical significance was set at a value of *p* < 0.05. These analyses were performed using the SPSS statistical package and the GraphPad Prism 5 software (San Diego, CA).

## 3. Results

### 3.1. Alloxan-Induced Diabetes in Rats

**Figure 1:**
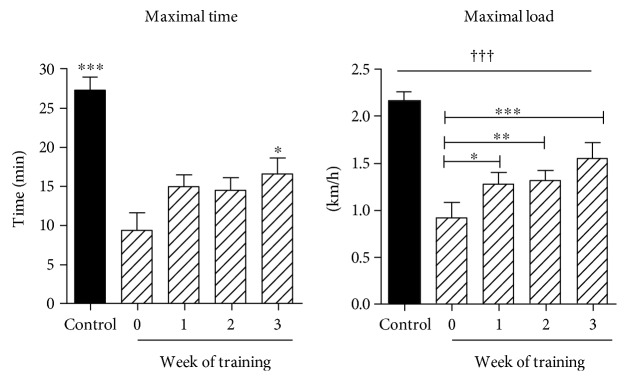
High-intensity training. Maximal time (min) and velocity (km/h) test. Rats were submitted to an exercise test before (control) and after induction of diabetes. Week 0 corresponds to the time point after the confirmation of diabetes. From week 0, the result of each week test was used to calculate the 80% of maximal capacity for the next week training. Then, every week, a maximal capacity test was performed. ^†††^*p* < 0.005 versus all the other groups; ^∗∗∗^*p* < 0.005 versus week 0; ^∗∗^*p* < 0.001 versus week 0; ^∗^*p* < 0.05 versus week 0.

Alloxan injection induced hyperglycemia in rats as expected for a type 1 model of diabetes (Table 1). Control animals increased body weight while both diabetic groups showed a slight weight reduction. All the animals in the training group completed the protocol and increased significantly their aerobic capacity (Figure 1). Diabetic rats submitted to the exercise protocol showed reduced plasma glucose levels compared to the sedentary diabetic rats (*p* < 0.05).

Exercise training tests at baseline (before diabetes) for maximal velocity and maximal time were performed. These tests were repeated after the induction of diabetes (week 0) and with these values was estimated the 80% of maximal performance that were used the next week of training for each animal. In the subsequent weeks, this procedure was repeated. In this way, diabetic rats increased their exercise capacity significantly (see Figure 1()), specially the maximal velocity that increased each week, although they did not reach the baseline values (before diabetes). Nevertheless, at the end of the third and fourth week of training, the test maximal capacity (velocity and time) tests were not performed.

### 3.2. Cardiac Morphology

**Figure 2:**
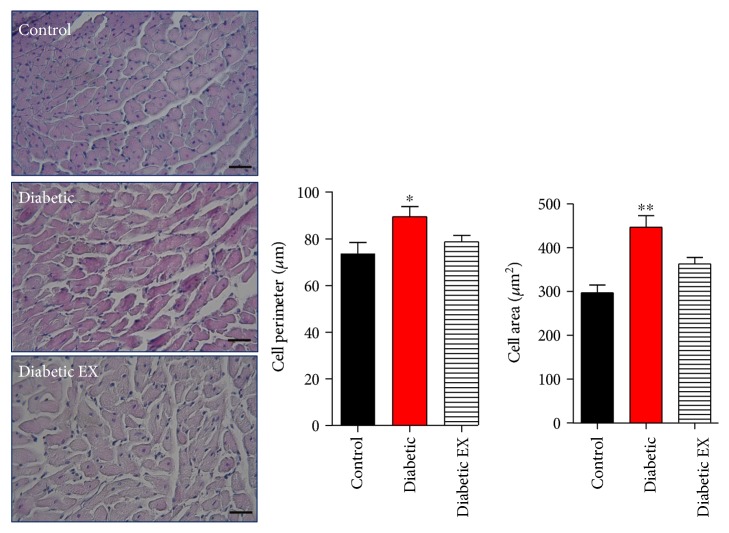
Cardiac hypertrophy induced by diabetes. Cardiac sections were stained with hematoxylin and eosin (see the photomicrographs). The bar graphs depict the statistical analysis for cellular hypertrophy (area and perimeter) in the control (nondiabetic), diabetic, and diabetic + exercise (diabetes EX) rats. The bar indicates 50 *μ*m. ^∗^*p* < 0.05 versus the other groups; ^∗∗^*p* < 0.01 versus control. *N* = 5 hearts in each group.

As it has been previously described, as part of the remodeling process that takes place in the diabetic heart, diabetes induced cellular hypertrophy of cardiomyocytes (Figure 2). Compared to the hearts from control normoglycemic rats, diabetic rat hearts presented increased perimeter of cardiomyocytes: 73 ± 7 *μ*m in the control group and 89.5 ± 4.3 *μ*m in the diabetic group (*p* < 0.05), and this value was reduced in diabetic rats that underwent exercise: 78.7 ± 2 *μ*m. Consistent with this, myocyte area was increased in diabetic hearts compared to that in controls and reduced in animals that underwent physical training: 297 ± 17 *μ*m^2^ in control rats, 446 ± 26 m^2^ in diabetic rats, and 363 ± 14 *μ*m^2^ in the diabetic + exercise group (*p* < 0.05).

**Figure 3:**
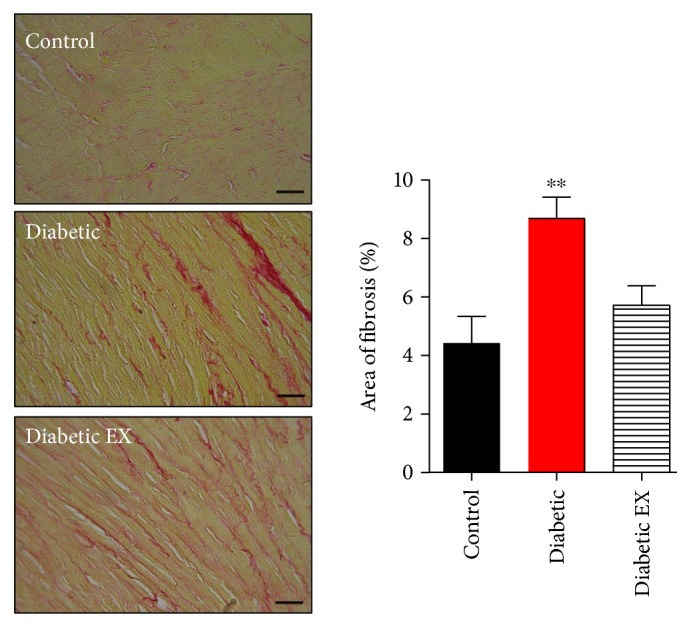
Cardiac fibrosis induced by diabetes. Cardiac sections were stained with Sirius red (see the photomicrographs). The bar graphs depict the statistical analysis for fibrosis in the control (nondiabetic), diabetic, and diabetic + exercise (diabetes EX) rats. *N* = 5 hearts in each group. The scale bar indicates 50 *μ*m. ^∗∗^*p* < 0.01 versus control.

As expected, diabetes induced an increase in fibrosis as collagen deposition in the heart as part of the cardiac damage (Figure 3). Nevertheless, chronic exercise reduced cardiac fibrosis: 4.43 ± 0.9% of fibrosis in the control group, 8.68 ± 0.7% in diabetic rats, and 5.72 ± 0.7% diabetic hearts from the rats that underwent high-intensity exercise.

### 3.3. Apoptosis

**Figure 4:**
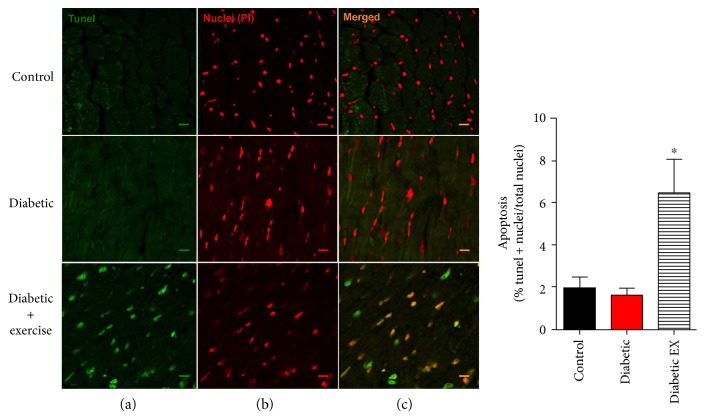
Cardiac apoptosis. Assessment of apoptosis by TUNEL. (a) Photomicrographs (green channel) show the positive nuclei for the TUNEL reaction of the representative cardiac sections from the control (nondiabetic), diabetic, and diabetic + exercise rats, *n* = 5 hearts in each group. (b) Photomicrographs (red channel) show total nuclei of the same sections stained with propidium iodide. (c) Photomicrographs show the merged signal for both channels. The scale bar indicates 10 *μ*m. ^∗^*p* < 0.05 versus the other groups.

It has been observed that diabetes is associated with increased degree of apoptosis in the myocardium [31]. For this reason, we investigated this type of cell death in our experimental animals, using the TUNEL assay (Figure 4). In the control normoglycemic and diabetic rats, the level of apoptotic index was low: 1.9 ± 2.2 and 1.5 ± 2%, respectively. Unexpectedly, this level was increased substantially in the diabetic + exercise group: 6.5 ± 10.2% (*p* < 0.05). These data suggest that high-intensity exercise training induced an increase in cardiomyocytes apoptotic death.

### 3.4. NADPH Oxidases

**Figure 5:**
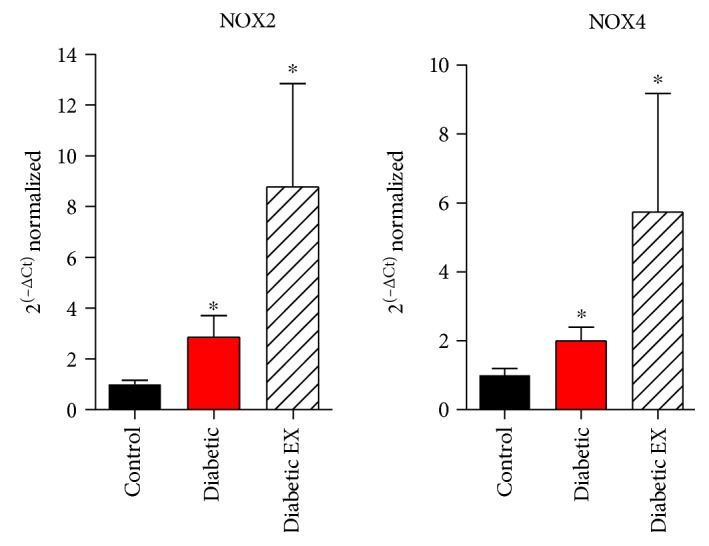
NOX2 and NOX4 mRNA expression in diabetic hearts. Quantification for NOX2 and NOX4 mRNA, normalized to the levels of GAPDH, by real-time PCR, from the control (nondiabetic), diabetic, and diabetic + exercise (diabetic EX) rat hearts; *n* = 3 hearts in each group. ^∗^*p* < 0.05 versus control.

Diabetes is associated with an increase in oxidative stress in the myocardium, particularly derived from NADPH oxidases as source of reactive oxygen species [32]. For this reason, we first looked into the expression at the level of NOX2 and NOX4 mRNA (Figure 5), the two main isoforms of NADPH oxidases that are expressed in the heart and, specifically, in the cardiac myocyte. Diabetes induced a twofold increase in the NOX2 mRNA level, compared to control normoglycemic animals (*p* < 0.05). Unexpectedly, exercise increased this level about eightfold. In the case of NOX4, diabetes also increased the mRNA levels (*p* < 0.05), and in the diabetes + exercise group, this increase was even more pronounced, although with substantial variability.

**Figure 6:**
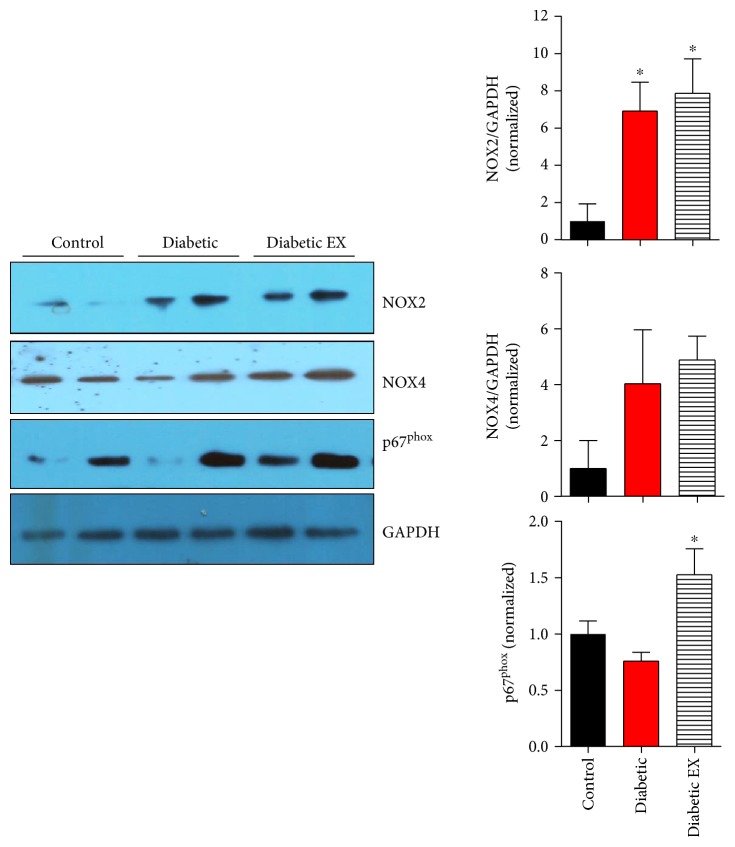
NOX proteins expression in diabetic cardiomyopathy. Representative Western blots for NOX2, NOX4, and p67^phox^ in the control (nondiabetic), diabetic, and diabetic + exercise (diabetic EX) rat hearts. The bar graphs indicate the densitometry analysis for 5 hearts of each group, normalized by GAPDH levels. ^∗^*p* < 0.05 versus control.

Next, we tested whether this increase in the NOX mRNA levels was followed by increased protein expression. For this purpose, we performed SDS-PAGE and Western blotting assays of cardiac samples (Figure 6). In the case of the protein levels of NOX2, both the diabetes and diabetes + exercise groups showed about a sevenfold increase. In the case of NOX4, there was a trend of increase but not statistically significant. On the contrary, the levels of p67^phox^, one of the regulatory subunits of NOX2, also showed a significant increase in the diabetic + exercise group (*p* < 0.05). These data suggest that the components of NOX2 are upregulated, increasing the levels of myocardial oxidative stress, especially in the group submitted to high-intensity exercise training.

### 3.5. Nitric Oxide Synthase

**Figure 7:**
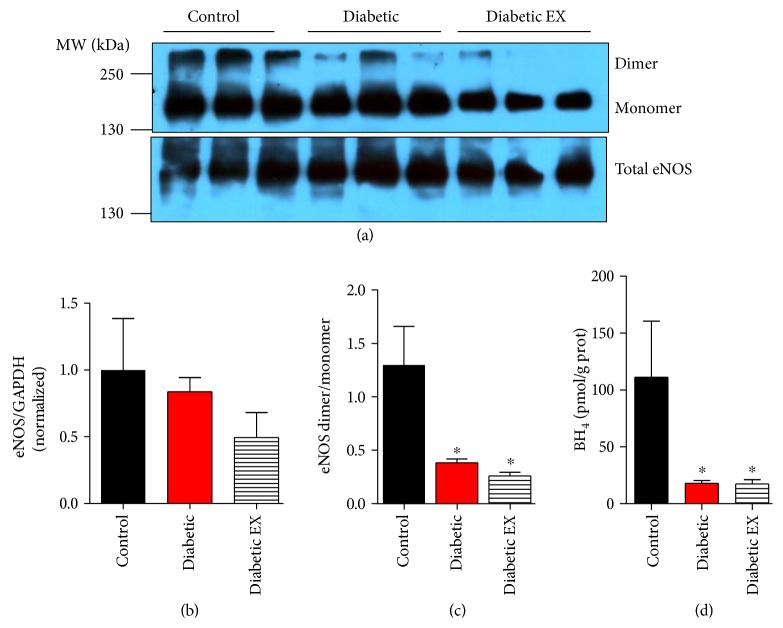
eNOS uncoupling in diabetes. (a) Representative Western blot for eNOS as dimer and monomer, and total cardiac eNOS from control (non-diabetic), diabetic and diabetic + exercise rats hearts (diabetic EX), *n* = 5 hearts each group. (b) Quantification for cardiac levels eNOS. (c) Ratio of dimer to monomer eNOS. (d) Intracardiac levels of tetrahydrobiopterin (BH_4_). ^∗^*p* < 0.05 versus control.

Next, we examined the nitric oxide pathway, which has been shown to be cardioprotective. First, we evaluated the cardiac levels of the endothelial nitric oxide synthase, by Western blotting (Figure 7). Neither diabetes nor exercise induced changes in the levels of eNOS (*p* = 0.4139).

A direct consequence of the increased production of reactive oxygen species (ROS) is the uncoupling of nitric oxide synthase. The oxidation of BH_4_, an essential cofactor for NOS activity, leads to the dissociation of NOS as a dimer into monomers. We quantified the levels of eNOS as a dimer and a monomer by Western blot under nonreducing conditions (Figure 7). This analysis revealed that diabetes induced strong eNOS uncoupling, increasing the formation of its monomer. High-intensity exercise was unable to restore eNOS as dimer. The eNOS dimer/monomer ratio was 1.3 ± 0.4 in the control group, 0.38 ± 0.04 in the diabetic group, and 0.26 ± 0.03 in the diabetic + exercise group (*p* < 0.05). Furthermore, exercise was unable to restore the intracardiac levels of BH_4_, an essential cofactor for NOS activity, that were reduced in diabetic rats: 111.1 ± 49.2 pmol/g protein in the control group, 17.9 ± 2.6 in the diabetic group, and 17.4 ± 3.7 in the diabetic + exercise group (*p* < 0.05). This result explains that both diabetic groups showed increased eNOS as monomer, in the uncoupled state. In most samples, BH_2_ levels were undetectable, for this reason, results are expressed as BH_4_ normalized to protein concentration, instead of a ratio BH_4_/BH_2_.

### 3.6. Calcium Handling Proteins

**Figure 8:**
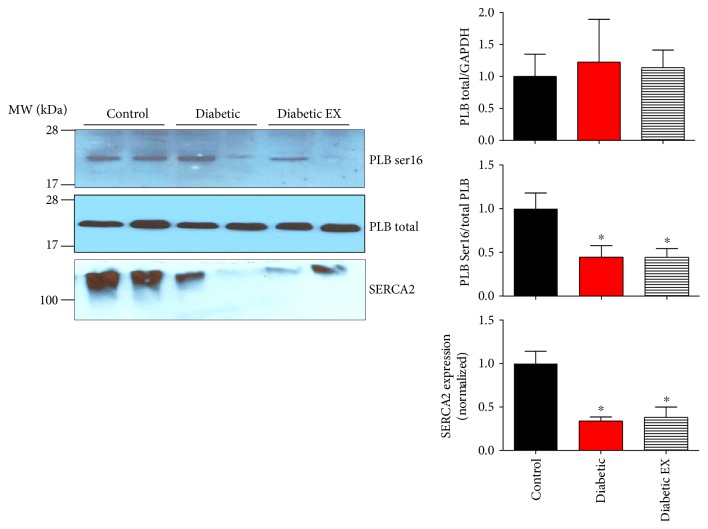
Phospholamban phosphorylation and SERCA2 expression. Levels of phospholamban (PLB) phosphorylation in control (non-diabetic), diabetic, and diabetic rats submitted to exercise training (diabetic EX). Representative Western blots for the phosphorylated form of phospholamban at serine 16 (PLB Ser16), total phospholamban (PLB), and SERCA2 from cardiac homogenates. The bar graphs correspond to the densitometry analysis for 5 hearts in each group. ^∗^*p* < 0.05 versus control.

Finally, we evaluated the level of Ca^2+^ handling proteins, to gain information regarding the status of the excitation contraction coupling elements in the diabetic hearts. Phospholamban (PLB) is a negative regulator of the sarcoplasmic calcium pump SERCA2 [33]. When PLB phosphorylated at serine 16 (by protein kinase A), it unleashes SERCA2 from its inhibition, being able to increase the Ca^2+^ reuptake to the sarcoplasmic reticulum. While the total levels of PLB were not altered by diabetes (Figure 8), the degree of PLB phosphorylation at serine 16 was reduced in diabetic animals and was not recovered by exercise (*p* < 0.05), suggesting impaired intracellular Ca^2+^ handling. We also assessed the levels of SERCA2. Both diabetic groups presented decreased levels of SERCA2 compared to nondiabetic rats (*p* < 0.05), which is associated with cardiac dysfunction, as the capacity to store Ca^2+^ in the sarcoplasmic reticulum is reduced (systolic function), and the velocity of relaxation is reduced (diastolic function).

## 4. Discussion

Exercise appears as an effective strategy for diabetic patients and has been recommended in moderate and high intensity [15]. In this study, we evaluated the effects of a high-intensity physical exercise training protocol on cardiac remodeling and myocardial proteins involved in the nitroso-redox balance in an animal model of type 1 diabetes. We found that this training program had a positive impact on cardiac remodeling, evidenced as reduction in myocyte hypertrophy and reduced collagen deposition (fibrosis), but was unable to restore the nitroso-redox environment. NADPH oxidase 2 system (NOX2) was upregulated; there were no changes in eNOS levels, but the degree of eNOS uncoupling was increased, being a potential additional source of superoxide. Furthermore, this probably increase in oxidative stress was associated with increased degree of cardiac apoptosis in diabetic animals submitted to the exercise program and reduced phospholamban phosphorylation. These data also suggest that the reverse remodeling process may be independent of increased oxidative stress. For example, it has been suggested that moderate-intensity training decreases myocyte cross-sectional area [24]. Interestingly, the high-intensity training increased the degree of apoptosis. This is contrary to what has been observed in rats under low-intensity exercise [18], which reduced the apoptotic index associated with a recovery in the level of antioxidant enzymes glutathione peroxidase, catalase, and superoxide dismutase. Nevertheless, in that study, plasma levels of glucose reached about 300 mg/dL in the diabetic animals, much lower compared to those in our study (around 600 mg/dL in the diabetic rats). In another recent study, Gimenes et al. in a similar low-intensity exercise protocol also observed a recovery in the cardiac levels of catalase and glutathione peroxidase, with glucose plasma levels around 500 mg/dL (similar to this study) [34].

Regarding the NADPH oxidase system, diabetes induced an increase in both NOX2 and NOX4 mRNA that was not reversed by exercise. Furthermore, NOX2 protein and p67^phox^, one of its regulatory subunits, were also increased in diabetes and not reversed by exercise, strongly suggesting an increased ROS production.

In a recent report, Sharma et al. described that in a protocol of exercise similar to ours, they observed a reduction in the levels of the mRNA (although not measured by quantitative PCR) and protein of p47^phox^ and p67^phox^, the regulatory subunits of NOX2, in the left ventricle of diabetic rats submitted to exercise training [35]. But again, the plasma glucose levels in diabetic animals attained about 300 mg/dL, which appears to be in important factor on the observed results.

It is possible that the reverse remodeling observed in the exercise-trained rats may be independent of oxidative stress but dependent, for example, on reverse of metabolic pathways such as nonoxidative glucose pathways [26]. In our study, exercise reduced the plasma levels of the advanced glycation end-products (AGEs, data not shown) in the diabetic animals. On the other hand, NOX-derived ROS may have induced signaling pathways converging, for instance, in increased autophagy [36, 37]. Autophagy is a homeostatic process important during stress conditions, aimed to obtain energy and amino acids from the degradation of proteins and organelles [38]. ROS produced by NOX2 and 4 may increase the process of autophagy in the diabetic heart. This increased autophagic flux may be beneficial for the diabetic heart [39].

Interestingly, our study showed an increase in the degree of uncoupling of the enzyme, associated with a reduction of the NOS cofactor BH_4_. It is possible that the increased oxidative stress derived from NOX2 and other sources may have been responsible for the reduced levels of BH_4_, since this molecule has been shown to be redox sensitive [40, 41].

Strategies to recover cardiac BH_4_ in diabetic animals have shown promising results reducing remodeling and improving ventricular function [11, 13]. Farah et al. showed that a high-intensity exercise increased the degree of eNOS dimerization [42]. Nevertheless, this increase was lost upon ischemia and even reduced upon reperfusion. This reinforces the concept that eNOS uncoupling is induced by oxidative environments, as those observed in cardiovascular diseases and diabetes [43–45].

In addition, it has been shown that NOS activity is related to phospholamban phosphorylation [46], a key protein in the regulation of the excitation-contraction coupling, as a regulator of SERCA2. On the other hand, it has been reported that diabetes reduces phospholamban phosphorylation [33, 46], in agreement with our observations. Exercise was unable to restore this phosphorylation, probably reducing the capacity of the sarcoplasmic reticulum for Ca^2+^ reuptake, along with the reduced levels of SERCA2. Nevertheless, in the diabetic heart, other abnormalities in the excitation-contraction coupling machinery are also present, like the ryanodine receptor and the Na/Ca exchanger [47–49].

Another possibility is that exercise training may have reduced inflammatory mediators in the diabetic myocardium. da Silva et al. found in rats treated with streptozotocin that a low-intensity swimming training reduced intramyocardial levels of TNF-alpha associated with a reverse remodeling of the diabetic hearts [50].

## 5. Conclusions

In conclusion, the present results suggest that high-intensity exercise is able to reverse cardiac remodeling in the diabetic heart but is unable to restore the nitroso-redox imbalance imposed and observed in this condition. This later could by restored by pharmacological manipulations that may include antioxidants and tetrahydrobiopterin.

## Figures and Tables

**Table 1:** Body weight and plasma glucose.

Parameters	Control	Diabetic	Diabetic + exercise	*p* value
Baseline body weight, g	307.5 ± 7.5	333.0 ± 8.6	335.0 ± 10.6	0.1258
Final body weight, g	452.5 ± 19.3	318.0 ± 6.2^†^	298.0 ± 26.1^†^	0.0004
Baseline plasma glucose (mg/dL)	87.0 ± 8.7	78.0 ± 2.0	100.6 ± 4.5	0.0501
Plasma glucose at the beginning of training (mg/dL)	78.7 ± 3.5	573.5 ± 44.1^†^	516.6 ± 72.7^†^	<0.0001
Plasma glucose at the end of training (mg/dL)	108.4 ± 5.8	587.9 ± 15.9^†^	476.7 ± 42.6^††^	<0.0001

^†^ *p* < 0.005 versus control; ^††^*p* < 0.05 versus diabetic.
